# Exploring Suitability of *Salsola imbricata* (Fetid Saltwort) for Salinity and Drought Conditions: A Step Toward Sustainable Landscaping Under Changing Climate

**DOI:** 10.3389/fpls.2022.900210

**Published:** 2022-06-08

**Authors:** Hasnain Alam, Muhammad Zamin, Muhammad Adnan, Adnan Noor Shah, Hesham F. Alharby, Atif A. Bamagoos, Nadiyah M. Alabdallah, Saleha S. Alzahrani, Basmah M. Alharbi, Shah Saud, Shah Hassan, Shah Fahad

**Affiliations:** ^1^Department of Biological Sciences, International Islamic University, Islamabad, Pakistan; ^2^Department of Biology, College of Sciences, United Arab Emirates University, Al Ain, United Arab Emirates; ^3^Department of Agriculture, University of Swabi, Swabi, Pakistan; ^4^Department of Agricultural Engineering, Khwaja Fareed University of Engineering and Information Technology, Rahim Yar Khan, Pakistan; ^5^Department of Biological Sciences, Faculty of Science, King Abdulaziz University, Jeddah, Saudi Arabia; ^6^Department of Biology, College of Science, Imam Abdulrahman Bin Faisal University, Dammam, Saudi Arabia; ^7^Biology Department, Faculty of Science, University of Tabuk, Tabuk, Saudi Arabia; ^8^College of Life Science, Linyi University, Linyi, China; ^9^Department of Agricultural Extension Education and Communication, The University of Agriculture, Peshawar, Pakistan; ^10^Hainan Key Laboratory for Sustainable Utilization of Tropical Bioresource, College of Tropical Crops, Hainan University, Haikou, China; ^11^Department of Agronomy, The University of Haripur, Haripur, Pakistan

**Keywords:** biochemical, drought, landscaping, native plants, salinity, physiological

## Abstract

In context of the climate change, major abiotic stresses faced by plants include salt stress and drought stress. Though, plants have similar physiological mechanisms to cope with these salt and drought stresses. The physiological and biochemical response of native plants to the combined application of salinity and drought stresses are still not well-understood. Thus, to investigate the combined effect of salinity and drought stresses, an experiment was conducted on *Salsola imbricata* with four levels of salinity and four drought intensities under the arid climatic conditions. The experiment was conducted in a randomized complete block design with a split-plot arrangement replicated three times. *S. imbricata* had been found resistant to different levels of individual and combined salt and drought stresses. *S. imbricata* survived till the end of the experiment. Salt and water stress did not show any significant effects on shoot weight, shoot length, and root length. The drought stress affected the photosynthetic rate, ion uptake and leaf water potential. However, salt stress helped to counter this effect of drought stress. Thus, drought stress did not affect plant growth, photosynthesis rate, and ion uptake when combined with salt stress. Increased Na^+^ and Cl^−^ uptake under the salt stress helped in osmotic adjustment. Therefore, the leaf water potential (LWP) decreased with increasing the salt stress from 5 dSm^−1^ until 15 dSm^−1^ and increased again at 20 dSm^−1^. At lower salt stress, ABA and proline content declined with increasing the drought stress. However, at higher salt stress, ABA content increased with increasing the drought stress. In conclusion, the salt stress had been found to have a protective role to drought stress for *S. imbricata*. *S. imbricata* utilized inorganic ion for osmotic adjustment at lower salinity stress but also accumulate the organic solutes to balance the osmotic pressure of the ions in the vacuole under combined stress conditions. Due to the physical lush green appearance and less maintenance requirements, *S. imbricata* can be recommended as a native substitute in landscaping under the salt and drought stresses conditions.

## Introduction

Agriculture is facing serious threats from abiotic factors such as salt stress and drought stress (Wang et al., 2003). Environmental fluctuations are rapidly increasing including salt and drought stresses, limiting plant productivity by 10% of arable land and more than 50% of major crops (Bartels and Sunkar, 2005). Globally, salt stress is affecting more specifically the irrigated agricultural land, while drought is severely affecting the agricultural crops (Zhu, 2002; Zamin et al., 2019a). Therefore, studies on the plants' response to salt and drought stresses are of primary importance. Halophytes have the ability to withstand and even benefit from salt and drought stresses conditions, which are lethal to the cultivated crops (Zamin and Khattak, 2018). Studying halophytes can lead to produce salt-tolerant crops through genetic modification and effective breeding (Ben Amor et al., 2005). These studies may also help to develop sustainable arid landscapes with native plants, which can conserve drought resources used for landscape irrigations (Zamin et al., 2018).

The effect of abiotic stresses including salt and water stresses are often indistinguishable and interconnected. For example, salt and drought stresses disrupt homeostasis and ion distribution resulting from osmotic stress in the cell (Wang et al., 2003). The plants physiological mechanisms to cope with salt and drought stresses are similar up to some extent. The water potential under salinity and drought decreases significantly in the similar pattern because under salt stress the plants' available water is also decreasing (Hasegawa et al., 2000). The simultaneous incidence of different stresses has positive or negative impacts on plant performance, depending on the nature and duration of the stresses (Niinemets, 2010). With the help of cross-tolerance, the plants have developed some special mechanisms to adapt to one stress and become resistant to some other stresses. This phenomenon is still an important challenge for the researchers and the exact mechanism of cross-tolerance is still not well-understood. Scientists are still focusing to get stable multiple stress tolerant traits in agronomical crops to improve yield, particularly in xeric conditions (Bahmani and Maali-Amiri, 2017).

The photosynthesis is a primary process that is influenced by the salt and drought stresses because of stomatal closure and decreasing the net CO_2_ diffusion to the chloroplast (Gibberd et al., 2002; Tezara et al., 2002; Anjum et al., 2011). However, the salt or drought stress tolerance is mainly associated with the maintenance of the net photosynthetic rate (Kumar et al., 2000; Anjum et al., 2011). The leaf Na^+^ and Cl^−^ concentrations increase significantly with increasing electrical conductivity (EC) of the irrigation water (Niu et al., 2012). The production of reactive oxygen species (ROS) is significantly increased under the salt and drought stresses (Miller et al., 2010). The ROS are generated during the stress metabolism as a toxic by-product and play an important role in signal transduction molecules during the plant responses to abiotic stresses (Miller et al., 2008). The overproduction of these ROS can cause oxidative damage to plants (Smirnoff, 1998). Plants have developed antioxidant defense mechanism, which can detoxify the adverse effect of ROS (Caverzan et al., 2012) and protect plant cells from oxidative damage by scavenging of ROS (Gill and Tuteja, 2010). Mostly, the researchers have investigated the responses of cultivated crops to salt and drought stresses on molecular levels (Umezawa et al., 2004). The ROS scavenging capacity of cultivated plants has been widely investigated by applying different stresses separately (Sekmen et al., 2014).

Native plants have the potential not only resist to the aforementioned stresses but also to provide many ecological benefits (Alam et al., 2017; Zamin et al., 2019b). Compared to cultivated relatives, native species have more ability to grow under the salt and drought stresses conditions (Morales et al., 2001; Fiedler, 2006; Stephens et al., 2006; Ochoa et al., 2009; Zamin and Khattak, 2017). According to Garci et al. (2004), many taxa are categorized as drought-resistant often based on the anecdotal observations. The physiological and molecular response of native plants to the combined application of salinity and drought stress is still not well-understood (Harb et al., 2010).

*Salsola imbricata* (Forssk.) (Arabic name: غضرب) belonging to the family *Amaranthaceae* is a perennial halophytic shrub that grows in deserts and arid regions of the Arabian Peninsula, southwestern Asia and North Africa. *S. imbricata* can also be used as a model plant to study the cross-tolerance for salt and drought stress and improve the stress resistance in many other plant species. Moreover, studying desert plants like *S. imbricata* for their field performance under the xeric conditions will provide guidelines for their proper maintenance in landscapes. We evaluated the field performance of *S. imbricata* under combined salt and drought stresses condition and study underlying stress resistance mechanism to overcome these stresses. Therefore, the study aimed to explore the suitability of *S. imbricata* for urban landscaping and to bring sustainability in landscaping.

## Materials and Methods

### Research Site

The field experiment to study the eco-physiological response of *S. imbricata* to different salt and drought stresses was carried out at the AL-Foa Research Farm, United Arab Emirates University, Al Ain, Abu Dhabi, UAE (24°12' N and 55°44' E) during 2015–2016. The experimental site was situated in the arid region, having a long hot summer season of 4 months, i.e., from May to September with a maximum temperature above 45°C. The winter prevails from mid-November to the end of February followed by a short spring season from March to April. The mean annual temperature varies between 12 and 45°C during winter and summer seasons, respectively (Statistics Center Abu Dhabi, 2015). The soil used in potting mix was sandy in nature, which was comprised of 87.5% sand, 5% silt, and 7.5% clay. More detailed soil properties are presented in Table 1 (Abdelfattah et al., 2009).

**Table 1:** Physicochemical properties of planting medium.

**Soil properties**	
**Texture**	
Sand (%)	87.50
Silt (%)	5.00
Clay (%)	7.50
Total carbonate (%)	24.53
EC (dSm^−1^)	9.49
pH	7.58
**Cations mg/kg**	
Ca	25.00
Mg	34.20
Na	53.80
K	7.52
**Anions mg/kg**	
Cl	46.80
HCO3	20.40
SO4	0.64
Mg:Ca Ratio	1.37

### Experimental Design

*Salsola imbricata* seeds were sown in germinating trays with growing media of potting soil and sweet sand 1:1 by volume. The soil used in potting mix was sandy in nature having 24.53% carbonate content with pH 7.58 and EC 9.49. The Ca^+^ content of the soil sample was 25 mg/kg whereas Mg was 34.2 mg/kg and low K^+^ content, i.e., 7.53 mg/kg (Table 1). After 3 weeks of germination, seedlings were transplanted to pots with 20 cm diameter and 15 cm height filled with sweet desert sand which has lower EC values and is considered good for agriculture purposes. Seedlings were thinned to one seedling per pot. After 1 month of transplantation of seedlings from germination trays to the pots, four saltwater treatments were prepared by dissolving NaCl in freshwater supplied by Al- Ain in municipality, i.e., 5 dS m^−1^ (Control; S1), 10 dS m^−1^ (low salinity level; S2), 15 dS m^−1^ (moderate salinity level; S3), and 20 dS m^−1^ (high salinity level; S4; Al-Dakheel et al., 2015; Zamin et al., 2019a). Salinity treatments were prepared in four different water tanks. These water tanks were connected to the drip irrigation line to supply water to each pot individually with four irrigation intensities. To estimate the Field capacity, the fully water-saturated soil was weighed and then dried to constant weight at 105°C. The weight difference between water-saturated and oven-dried soil was taken as the weight of water needed to bring soil to field capacity and lower FC was calculated accordingly. Four irrigation intensities were: 100% field capacity (Control; C), 80% field capacity (low stress), 60% field capacity (moderate stress), and 40% field capacity (severe stress) (Álvarez et al., 2009). Plants were irrigated 2–3 times per week, depending upon evaporative demand using the drip irrigation system with one emitter per plant each delivering 2 Lh^−1^. The amount of water applied to the control varied between 788 and 1,182ml per pot per week. The average of water was 985 ml/week for the WL1 (control) and 787, 590, and 392ml/week for WL2, WL3, and WL4, respectively. The NPK ^@^ 5–7 g/plant was applied to each plant before the start of experiment. Agronomic practices, e.g., weeding and crop maintenance, etc., were equally applied to all treatments during the entire growing period of plants. Experiment was conducted in open field and plants were grown under natural environmental conditions. The mean monthly temperature ranged between 32.9 and 33.4°C and humidity from 21 to 29% while 0mm rainfall was forecasted at the beginning and end of the experiment. The experiment was conducted in a randomized complete block design replicated three times. The salinity levels were allotted to the main plot while the irrigation intensities were allotted to the sub-plots.

### Percent Survival, Harvesting, and Sampling

Plants that survived under each stress treatment were counted and the survival percentage was calculated. Three plants from each treatment were harvested after 6 months of treatment application to record morphological parameters. Plant samples from each treatment were collected and instantly ground in liquid nitrogen and stored at −80°C for the quantitative chemical analysis.

### Morphological Traits

After harvest, the plant samples were carefully cleaned from sand, washed with distilled water, and dried with the help of tissue paper. After harvesting, each plant was divided into shoots and roots and root and shoot length were measured. Shoot length was measured from the base of stem till the apex end while root length was measured from the root base up to the end of primary root. Samples were oven-dried (60°C) for 24 h and weighted (±0.0001 g). For morphological traits, all the samples were put in Ziploc bags, placed in an ice bag at 4°C, and transferred to the laboratory.

### Physiological Traits

The photosynthetic rate of upper, lower, and basal leaves was measured weekly using a Plant Photosynthesis Meter (EARS, Netherlands; Samarah, 2005). Replicated leaf water potential (MPa) was recorded during midday using a WP4C Dewpoint psychrometer (Decagon Devices, Inc., USA; Xiong et al., 2014). Leaf water potential was recorded after 1 and 5 months of treatment application. Phosphorus concentrations were estimated in plant leaves at the end of the experiment by Olsen (1954) methodology. Na^+^ content (μ mole g^−1^) of plant extracts was determined by the Flame Emission Spectroscopy at the end of the experiment. For Cl^−^ content, 50mg of leaf and root samples were ground and heated in distilled water for 3 h (80°C). The Cl^−^ content (μmole g^−1^) of the extract was then determined with the chloride analyzer at the end of the experiment.

### Biochemical Traits

The ABA and proline extraction was performed on 10mg of freeze-dried leaf tissue as described by Forcat et al. (2008). The samples were analyzed for ABA and proline using LCMS/MS, and were filtered through a 0.45μm cellulose acetate syringe. The phytohormones separation was done using a C18 column (ZORBAX Eclipse Plus). An injection of 2 μl was loaded onto the C18 column (1.8μm particle size, 2.1mm inner diameter, and 50mm long) at a flow rate of 0.2 ml/min and the column temperature was kept at 35°C. The liquid chromatography was connected to an Agilent Technologies Mass Spectrometry (6420 Triple Quad detector). For elution, solvent A consists of formic acid (0.1%) with distilled water and solvent B consists of an LCMS grade acetonitrile were used. The analytical procedure was as follows.

Solvent A was used (5min), then the gradient from 0 to 100% solvent B was used (5–20min), after the solvent B was kept constant (5min) and at 25.1min solvent A was 100% was used for 30min. During the analysis with LC–MSMS only negative polarity mode was used for ABA and Proline analysis. For fragmentation, nitrogen gas was used. The capillary voltage was 4,000V, the gas flow was 8 L/min, the gas temperature was 300°C and the nebulizer pressure was 45 psi.

### Statistical Analysis

Two way analysis of variance (ANOVA) was used to check the effect of salinity, drought, and their interaction on morphological, biochemical, and physiological traits. While the normality was checked with Shapiro–Wilk test. The *post-hoc* Tukey HSD was used to check the comparison between treatments. All the analysis was performed using the SPSS software at 5% probability level.

## Results

### Percent Survival, Root and Shoot Length and Weight

Results concerning the morphological response of *S. imbricata* to varying salt and drought stresses are given in Table 2. The ANOVA revealed that all the morphological parameters (percent survival, root and shoot length and weight) did not show any significant (*p* > 0.05) effect by the drought stress. A similar response was found in the case of salinity stress except root weight (g) which was significantly affected by increasing salinity. The percent survival of *S. imbricata* did not affect significantly (*p* > 0.05) by different levels of salt and drought stresses. The interaction between salt and drought stresses was also not significant and *S. imbricata* survived on all salt and drought levels with percent survival >90% (Table 2). However, the root weight was significantly affected by the salt stress while the drought stress had no significant effect. The root weight was maximum under the lower salinity and decreased with increasing drought stress. The root weight (10.22 g) observed at 5 dS m^−1^ was statistically at par to the root weight (7.50 g) at 10 dS m^−1^. Furthermore, the root weight (4.87 and 9.69 g) recorded at 15 and 20 dS m^−1^ was statistically similar with root weight at 10 dS m^−1^.

**Table 2:** Morphological response of *S. imbricata* to varying drought and salinity stresses.

**Drought stress (% Field Capacity)**	**Survival percentage**	**Root length (cm)**	**Shoot length (cm)**	**Root weight (g)**	**Shoot weight (g)**
100 (WL1)	93.39	51.52	48.69	8.07	136.19
80 (WL2)	94.46	52.24	50.21	5.84	98.64
60 (WL3)	94.79	48.70	46.49	7.19	65.59
40 (WL4)	93.25	50.72	47.00	8.16	94.25
LSD (0.05)	NS	NS	NS	NS	NS
**Salinity stress (dS m** ^**−1**^ **)**					
5 (S1)	93.93	49.48	48.70	10.22a	101.90
10 (S2)	96.03	49.47	50.21	7.50ab	86.06
15 (S3)	91.39	50.28	45.52	6.68b	84.15
20 (S4)	93.54	53.97	47.96	4.87b	122.56
LSD	NS	NS	NS	3.45	NS
Interaction (WS*SS)	NS	NS	NS	NS	NS

Means with different letters in each category are significantly different at α = 0.05. NS, WS, SS, and LSD stand for no significant, drought stress, salinity stress and least significant difference, respectively. The * symbol indicates the interaction.

#### Photosynthetic Rate, Leaf Water Potential, Na^+^ Uptake, and Cl^–^ Uptake

Physiological response of *S. imbricata* to different drought and salinity stresses is shown in Table 3. According to the ANOVA, varying responses were recorded for different physiological traits. The drought stress did not show any significant (*p* > 0.05) effect on all the parameters except leaf water potential (MPa) which was significantly affected. In contrast to drought stress, salinity stress had a significant (*p* < 0.05) effect on all the physiological parameters of *S. imbricata* except photosynthetic rate. However, the interaction between salt and drought stresses was found significant.

**Table 3:** Physiological response of *S. imbricata* to varying drought and salinity stresses.

**Drought stress (% field capacity)**	**Photosynthetic rate (μmol m^−2^ S^−1^)**	**Leaf water potential (MPa)**	**Na^+1^ uptake (μ mole g^−1^)**	**Cl ^−1^ uptake (μ mole g^−1^)**
100 (WL1)	23.23	−21.99	405.9	40.42
80 (WL2)	21.24	−23.90	419.7	44.00
60 (WL3)	19.95	−28.94	423.8	43.33
40 (WL4)	17.55	−29.38	420.3	41.83
LSD (0.05)	NS	5.30	NS	NS
**Salinity stress (dS m^−1^)**				
5 (S1)	22.48	−20.30	398.8b	38.67
10 (S2)	19.90	−24.26	428.7a	42.33
15 (S3)	19.73	−35.01	404.3b	40.63
20 (S4)	20.17	−24.47	438.0a	47.96
LSD	NS	5.30	14.50	5.84
Interaction (WS*SS)	Figure 1	Figure 2	Figure 3	Figure 4

Means with different letters in each category are significantly different at α = 0.05. NS, WS, SS, and LSD stand for no significant, drought stress, salinity stress and least significant difference, respectively. The * symbol indicates the interaction.

**Figure 1:**
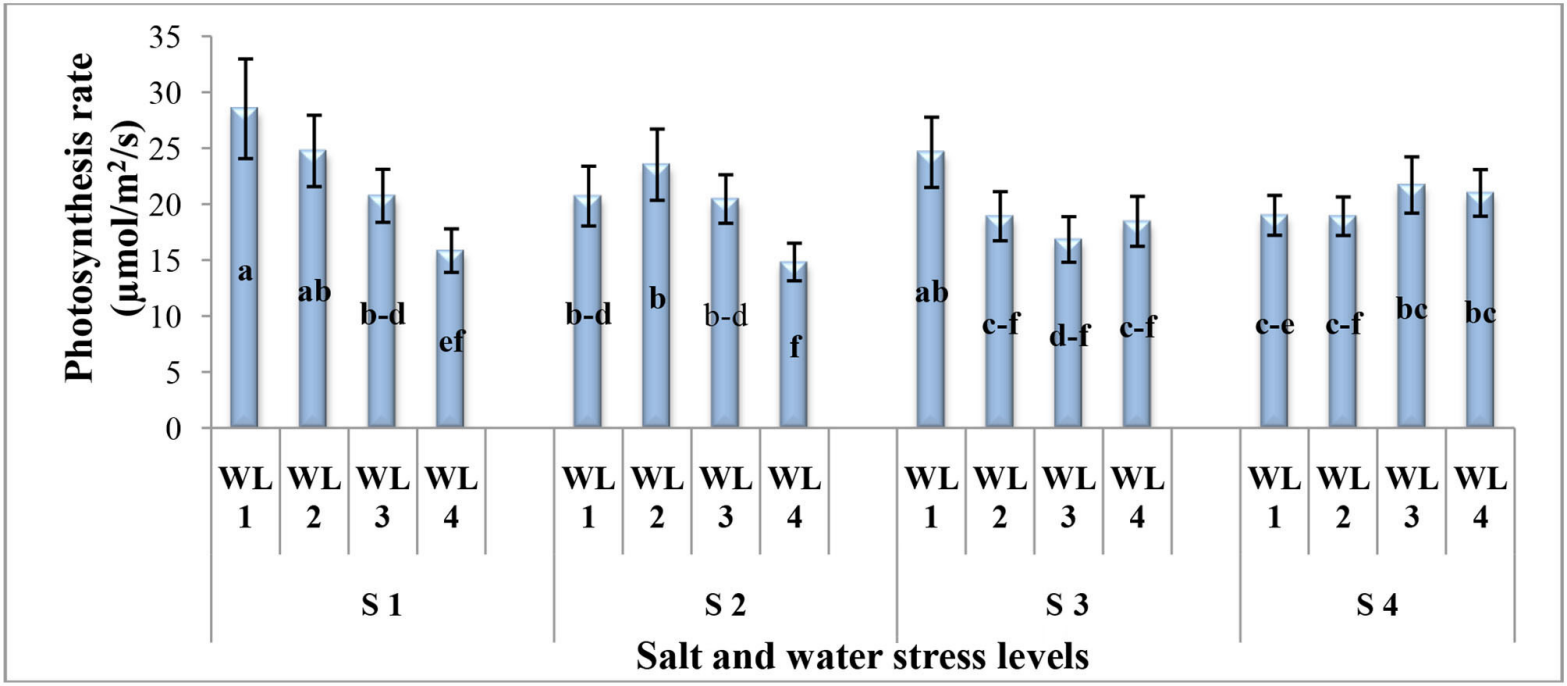
Interactive effect of salinity and drought stress on the photosynthesis rate (μmol m^−2^ S^−1^) of *S. imbricata*. Value bars with different letters are significantly different from each other at α = 0.05, while error bar represents the standard error of mean (*n* = 3). WL stands for water level (WL1 = 100% field capacity, WL2 = 80% field capacity, WL3 = 60% field capacity, and WL4=40% field capacity) while S represents salinity (S1 = 5 dS m^−1^, S2 = 10 dS m^−1^, S3 = 15 dS m^−1^, and S4 = 20 dS m^−1^).

The interactive effect of salinity and drought stress was found significant for photosynthetic rate (Figure 1). Generally, under the drought stress, the photosynthetic rate decreases with increasing salinity levels up to 15 dSm^−1^ while at 20 dS m^−1^, increasing tendency was found. The maximum photosynthetic rate was observed at 100% field capacity under 5 dS m^−1^ which was statistically similar to S1WL2 and S3WL1. It was evident that *S. imbricata* can maintain the photosynthesis under combine drought and salinity stresses to cope with the harsh and adverse climatic conditions.

**Figure 2:**
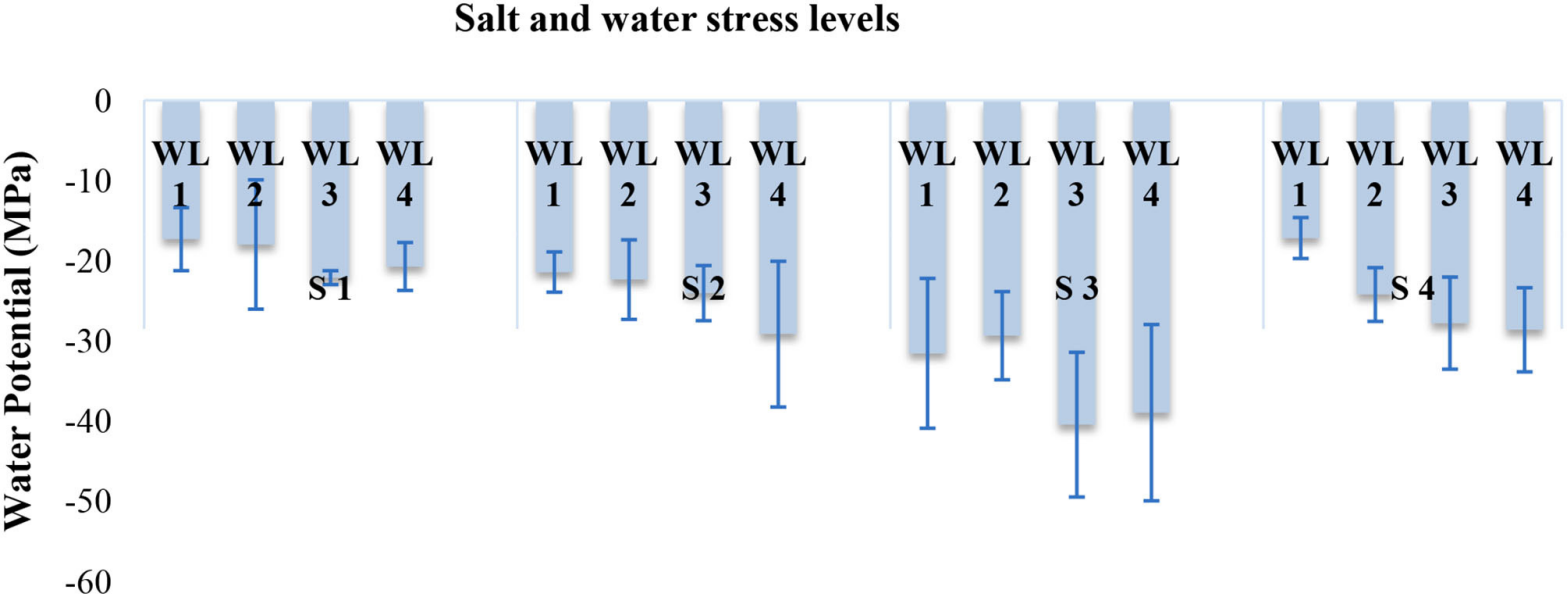
Interactive effect of salinity and drought stress on the water potential (MPa) of *S. imbricata*. Error bar represents the standard error of mean (*n* = 3). WL stands for water level (WL1 = 100% field capacity, WL2 = 80% field capacity, WL3 = 60% field capacity, and WL4 = 40% field capacity), while S represents salinity (S1 = 5 dS m^−1^, S2 = 10 dS m^−1^, S3 = 15 dS m^−1^, and S4 = 20 dS m^−1^).

Water potential was also significantly affected in response to the interaction of salinity and drought stress as indicated in Figure 2. Leaf water potential decreased with increasing salt stress from 5 dS m^−1^ until 15 dS m^−1^. However, at salinity stress of 20 dS m^−1^ leaf water potential was not much affected like 15 dSm^−1^. Increasing water stress also decreases the leaf water potential under all salt stress (Figure 2).

**Figure 3:**
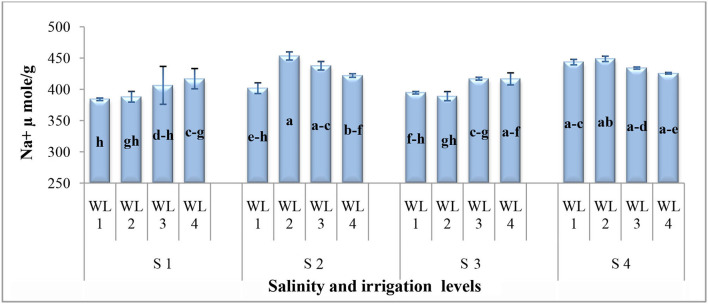
Interactive effect of salinity and drought stress on Na^+^ uptake (μmol g^−1^) of *S. imbricata*. Value bars with different letters are significantly different from each other at α = 0.05, while error bar represents the standard error of mean (*n* = 3). WL stands for water level (WL1 = 100% field capacity, WL2 = 80% field capacity, WL3 = 60% field capacity, and WL4 = 40% field capacity), while S represents salinity (S1 = 5 dS m^−1^, S2 = 10 dS m^−1^, S3 = 15 dS m^−1^, and S4 = 20 dS m^−1^).

**Figure 4:**
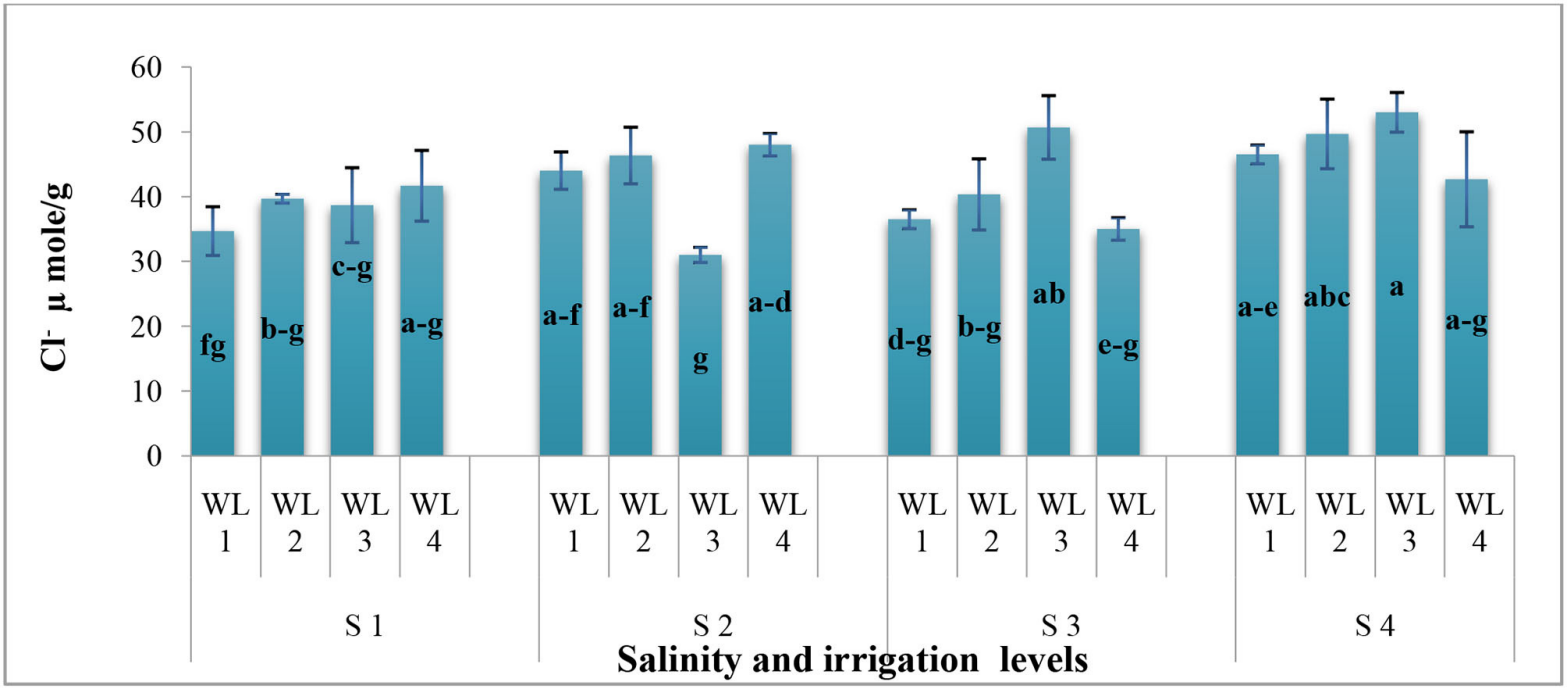
Interactive effect of salinity and drought stress on the Chloride uptake (μmol g^−1^) of *S. imbricata*. Value bars with different letters are significantly different from each other at α = 0.05, while error bar represents the standard error of mean (*n* = 3). WL stands for water level (WL1 = 100% field capacity, WL2 = 80% field capacity, WL3 = 60% field capacity, and WL4 = 40% field capacity), while S represents salinity (S1 = 5 dS m^−1^, S2 = 10 dS m^−1^, S3 = 15 dS m^−1^, and S4 = 20 dS m^−1^).

As far as the interactive effect of salinity and drought stress is concerned, the ion uptake (Na^+^ and Cl^−^ contents) was significantly affected by the salinity and drought stresses (Figures 3, 4). Na^+^ concentration had an interactive effect (*p* ≤ 0.05) on salt and water stress. The Na^+^ content increased with increasing the salt and water stress. Even at the low salt stress level, when external Na^+^ was low, Na^+^ concentration increased under the water stress in shoots (Figure 3). Generally, Cl^−^ content increases with increasing salinity. The Cl^−^ content increased with increasing the drought stress at lower salinity, while at higher salinity level, Cl^−^ content decreased after the drought stress reached to a certain level (Figure 4). This shows that *S. imbricata* has the ability to enhance the ion uptake under the severe drought and salinity conditions.

#### ABA (μg g^–1^ FW) and Proline (μg g^–1^ FW) Contents

Results concerning biochemical response of *S. imbricata* to varying drought and salinity stresses are presented in Table 4. The ANOVA revealed that ABA and proline content were significantly salt and drought stress interaction.

**Table 4:** Biochemical response of *S. imbricata* to varying drought and salinity stresses.

**Drought stress (% field capacity)**	**ABA (μg g^−1^ FW)**	**Proline (μg g^−1^ FW)**
100 (WL1)	134.3	7,138
80 (WL2)	100.2	6,324
60 (WL3)	107.4	2,674
40 (WL4)	91.7	4,313
LSD (0.05)	31.8	2,333
**Salinity stress (dS m^−1^)**		
5 (S1)	102.4	5,969
10 (S2)	105.6	5,453
15 (S3)	107.5	5,355
20 (S4)	118.1	3,672
LSD	NS	NS
Interaction (WS*SS)	Figure 5	Figure 6

Means with different letters in each category are significantly different at α = 0.05. NS, WS, SS, and LSD stand for no significant, drought stress, salinity stress and least significant difference, respectively. The * symbol indicates the interaction.

**Figure 5:**
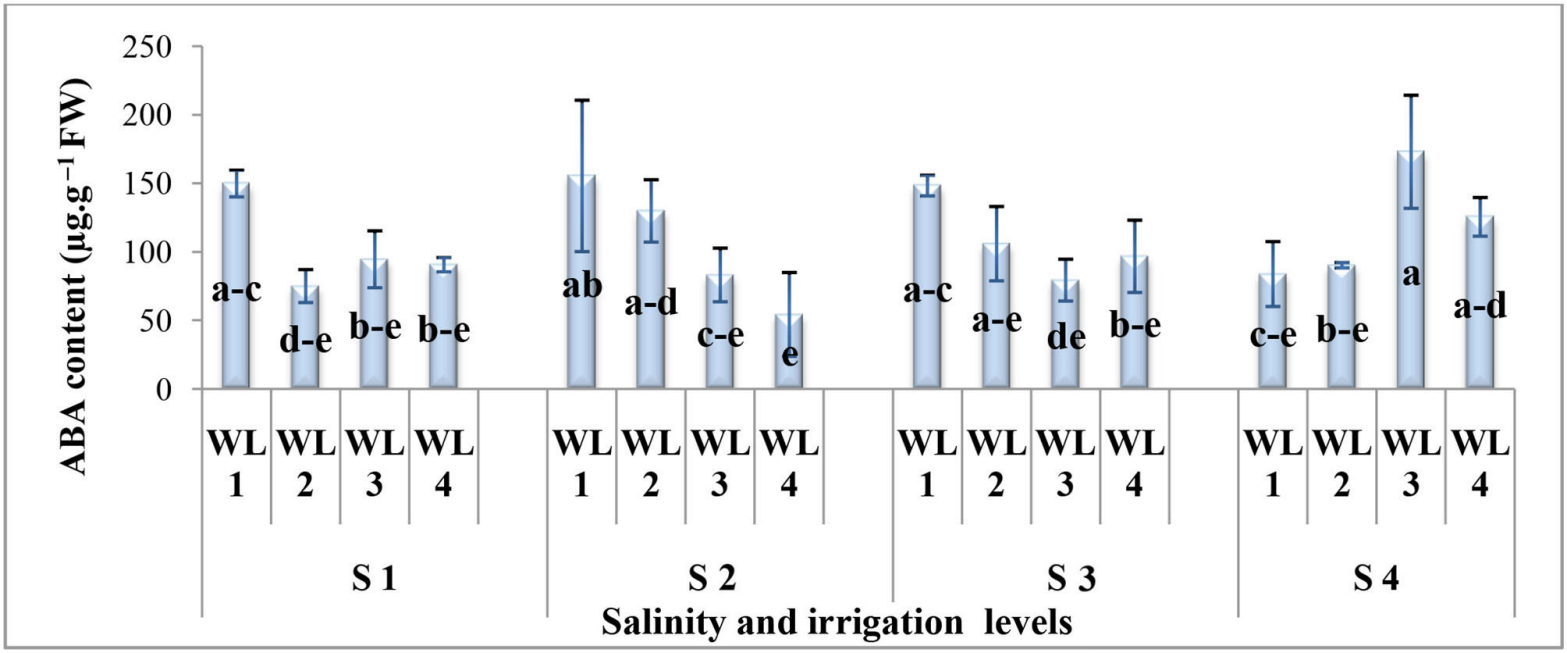
Interactive effect of salinity and drought stress on the ABA content (μg g^−1^ FW) of *S. imbricata*. Value bars with different letters are significantly different from each other at α = 0.05, while error bar represents the standard error of mean (*n* = 3). WL stands for water level (WL1 = 100% field capacity, WL2 = 80% field capacity, WL3 = 60% field capacity, and WL4 = 40% field capacity) while S represents the salinity (S1 = 5 dS m^−1^, S2 = 10 dS m^−1^, S3 = 15 dS m^−1^, and S4 = 20 dS m^−1^).

The interactive effect of salinity and drought stress was found significant for the ABA content (Figure 5). Generally, under combine salt and drought stress, the ABA content decreases with increasing drought stress for salinity levels up to 15 dS m^−1^ while at 20 dS m^−1^, the trend was the opposite. Under the salt stress of 20 dS m^−1^, maximum ABA production was recorded at 60% field capacity which is statistically at par with 40% field capacity. However, the ABA response to 60 and 40% field capacity was similar at all salinity levels. The maximum ABA content was observed at 60% field capacity under severe salinity (20 dS m^−1^) which was statistically similar to 100 and 80% field capacity at 10 and 15 dS m^−1^ and 100% field capacity at 5 dS m^−1^. It was evident that *S. imbricata* increased the ABA production under severe drought and salinity stress to combat their growth- and performance-related adversities.

**Figure 6:**
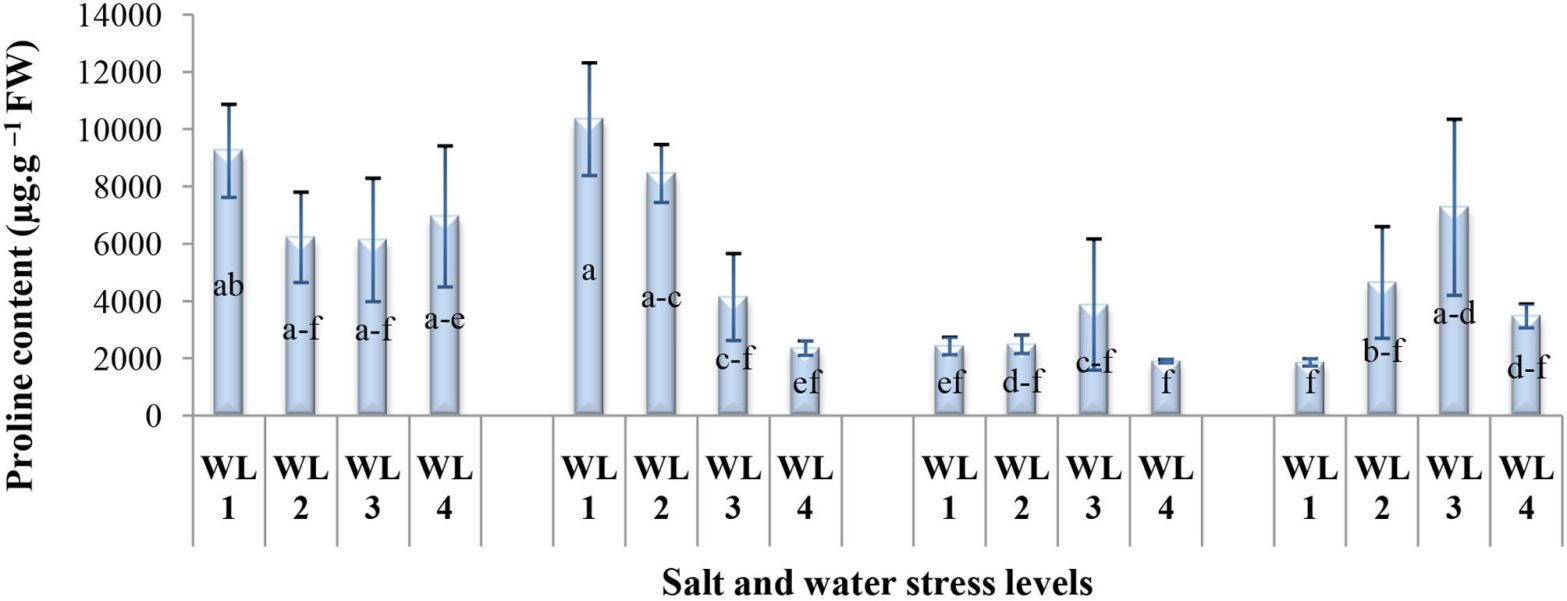
Interactive effect of salinity and drought stress on the proline content (μg g^−1^ FW) of *S. imbricata*. Value bars with different letters are significantly different from each other at α = 0.05, while error bar represents the standard error of mean (*n* = 3). WL stands for water level (WL1 = 100% field capacity, WL2 = 80% field capacity, WL3 = 60% field capacity, and WL4 = 40% field capacity), while S represents salinity (S1 = 5 dS m^−1^, S2 = 10 dS m^−1^, S3 = 15 dS m^−1^, and S4 = 20 dS m^−1^).

The proline content significantly varied in response to the interaction of salinity and drought stress as shown in Figure 6. The proline content decreases with increasing drought stress and salt stress together except S4. For salt stress of 20 dS m^−1^, proline content increased up to 60% field capacity and then declined. Maximum proline production was recorded 60% field capacity at 20 dS m^−1^ indicating that *S. imbricata* enhances the proline production under severe salinity and drought stress to cope with such stresses.

## Discussion

In the present experiment, *S. imbricata* survived for 6 months with no significant effect of salt and drought stress on the growth parameters. It is evident that *S*. *imbricata* is resistant to salt and drought stress.

Salt and drought stress are considered as separate and additive factors contributing to growth reduction (Munns, 2002; Chaves et al., 2009). However, in the current experiment, salt and drought stress did not show any significant effect on shoot weight, shoot length, and root length of *S. imbricata*. Higher salinity helped to reduce the negative effects of drought stress. Only root weight decreased with increasing salt stress. Moderate salinity (50–250mM NaCl) can stimulate the growth of many halophytes (Flowers et al., 1986; Khan et al., 2000). NaCl may have positive effects under the drought stress, i.e., salt stress alleviated the negative effects of drought stress. These positive effects of salinity were reported for *Atriplex nummularia* (Hassine et al., 2008), *Sesuvium portulacastrum* (Slama et al., 2007a), *A. canescens* (Glenn and Brown, 1998), *A. lentiformis* (Meinzer and Zhu, 1999), *Suaeda fruticosa* (L.) Forssk (Khan et al., 2000), and *A. halimus* (Alla et al., 2012). This improved plant performance under combined salt and drought stress may be due to their effect on osmotic adjustment through higher Na^+^ and proline accumulation and decrease of K^+^ accumulation (Wu et al., 2015).

Salt and drought stresses showed a significant interactive effect on the photosynthetic rate of *S. imbricata*. At lower salt stress, the photosynthetic rate decreased with increasing drought stress. On the other hand, at severe salt stress (20 dS m^−1^), the drought stress had no significant effect on the photosynthetic rate. The salt stress had a protective effect on the photosynthetic rate (Figure 1). The current results are in line with Wang et al. (2011) for *Tamarix chinensis* Lour and with Miranda-Apodaca et al. (2018) for quinoa. Under salt or drought stress, leaf water potential and thus photosynthetic activity is decreased (Razzaghi et al., 2011). This reduction in photosynthesis can be caused by a stomatal limitation with stomatal closure (Nicolas et al., 1993; De Pascale and Barbieri, 1995; Goldstein et al., 1996) non-stomatal limitation (disturbance of photosynthetic activity; Downton, 1977; Drew et al., 1990) or both limitations at low and high salt concentration (Downton et al., 1990; Yeo et al., 1991). The drought stress can inhibit the activity of photosystem II and the rate of CO_2_ assimilation (Bloch et al., 2006; Monti et al., 2006) which in turn could decrease the photosynthesis (Wu et al., 2016).

The Na^+^ and Cl^−^ uptake had significant results for the salt and drought stress interaction. Na^+^ and Cl^−^ uptake was significantly increased with increasing the salt stress. Drought stress was also found to increase Na^+^ and Cl^−^ uptake (Figure 3). However, *S*. *imbricata* decreased the Na^+^ uptake with increasing the drought stress at the highest salinity level (20 dS m^−1^). Studies carried out to evaluate the combined effects of salt and drought stress are in line with our findings (Martínez et al., 2005; Slama et al., 2008; Khalid and Cai, 2011; Khan et al., 2017a,b). Under saline conditions, Na^+^ in the growth medium might compete with K^+^ in the low absorption by the roots (Blumwald, 2000).

In response to low water potential under the salt and drought stress conditions additional solutes are accumulated which is referred as osmotic adjustment (OA; Zhang et al., 1999; Verslues et al., 2006). In halophyte species, Na^+^ presents in the vacuoles is involved in osmotic adjustment (Martínez et al., 2005; Slama et al., 2007b). Salt stress results in Na^+^ and Cl^−^ accumulation in shoots which are more effective for osmatic adjustment than the production of organic solutes under drought stress (Liu et al., 2008; Slama et al., 2008; Sucre and Suarez, 2011; Álvarez et al., 2012). Hassine et al. (2008) reported that during the stress period, shoot water potential remained lower in *Atriplex halimus* plants exposed to PEG than in those exposed to the highest dose of NaCl. Miranda-Apodaca et al. (2018) stated that plants under salt stress exhibit a greater capacity for osmotic adjustment while plants subjected to drought stress treatment showed more dehydration. Thus, the NaCl addition mitigated the deleterious impact of osmotic stress on growth ( Martínez et al., 2005 :Wu et al., 2016). Increasing salinity beyond toxic levels can be managed by halophytes using the strategy of exclusion of salts *via* salts glands present on their lower surface of leaves (Zamin et al., 2019a).

Abscisic acid accumulates and involves in all the aspects of the low water potential response. ABA-derived root growth and stomatal conductance are important in the avoidance of lower growth (Schroeder et al., 2001; Sharp and LeNoble, 2002; Verslues et al., 2006). As a dehydration avoidance response, ABA induces the accumulation of compatible solutes (Ober and Sharp, 1994). ABA production is a signal for the stomatal closure and reduction of stomatal density to decrease water loss by transpiration (Razzaghi et al., 2011; Adolf et al., 2013). ABA induced the stomatal closure by a reduction in the turgor pressure of guard cells (Schroeder et al., 2001; Bartels and Sunkar, 2005). These responses improve the water-use efficiency of the plant for the short term (Waseem et al., 2011; Oliveira et al., 2013). The NaCl stress did not affect transpiration or ABA levels of *Atriplex spongiosa* up to 75mol m^−3^ but transpiration fell and ABA levels rose when the NaCl was increased upto 150mol m^−3^. The drought stress resulted an increase in the leaf ABA content while salt stress had no effect (Achuo et al., 2006). A report by Li et al. (2011) stated that *Cotinus coggygria var. cinerea* significantly reduced the relative growth rate, but increased the endogenous ABA under drought.

Proline accumulation relates more to the osmotic stress than any specific salt effect (Munns, 2002). Martínez et al. (2005) reported that 0 or 15% PEG had no impact on the proline concentration at low NaCl (50mM) concentration (Martínez et al., 2005). *Atriplex spongiosa* and *Suaeda monoica* recorded low proline contents at 300 and 500mol m^−3^ NaCl or below, respectively. However, a significant increase was detected at high salinities (Storey and Jones, 1979). *Atriplex halimus* showed similar responses after treating seedlings with either 50, 300, and 550mM NaCl or drought (control and withholding water). Proline was significantly increased only by the high salt stress and drought stress, nonetheless, combined treatments led to decrease if any (Alla et al., 2012). This significant increase was still in low concentration which was supposed to function as osmoprotectant.

## Conclusion

Both salt and drought stress had no significant effect on the survival percentage and growth performance of *S. imbricata*. However, severe salt stress induced a decrease in root weight. Salt stress help to alleviate negative effects of drought stress through accumulation of Na^+^ and Cl^−^ ions and organic solutes at higher salinity. In conclusion, *S. imbricata* can be classified as the salt includer halophyte. This species achieved osmoregulation by adopting intracellular compartmentalization of ions and avoid high concentration of these ions in cytoplasm. It can be concluded that *S. imbricata* can survive under drought and saline conditions up to 20 dS m^−1^ without affecting growth and morphology. Therefore, it can be recommended as substitute in landscaping under extreme drought and saline conditions. Further studies can be carried out to study the economical uses of *S. imbricata* and determine the optimal salinity and irrigation requirements of *S. imbricata*. Furthermore, studies can be carried out to find out the molecular mechanisms that make *S. imbricata* resistance to salt and drought stress.

## Data Availability Statement

The raw data supporting the conclusions of this article will be made available by the authors, without undue reservation.

## Author Contributions

HAla and MZ: conceptualization. MA, AS, SS, SH, HAlh, AB, and NA: methodology and formal analysis. SA and MZ: writing—original draft preparation. BA and SF: writing—review and editing. MZ: supervision. HAlh, AB, NA, SA, and BA: funding acquisition. All authors contributed to the article and approved the submitted version.

## Funding

This project was funded by the Deputyship for Research and Innovation, Ministry of Education in Saudi Arabia under project number (IFPIP: 1132-130-1442).

## Conflict of Interest

The authors declare that the research was conducted in the absence of any commercial or financial relationships that could be construed as a potential conflict of interest.

## Publisher's Note

All claims expressed in this article are solely those of the authors and do not necessarily represent those of their affiliated organizations, or those of the publisher, the editors and the reviewers. Any product that may be evaluated in this article, or claim that may be made by its manufacturer, is not guaranteed or endorsed by the publisher.
